# Study of *Host-Guest* Interaction and In Vitro Neuroprotective Potential of Cinnamic Acid/Randomly Methylated β-Cyclodextrin Inclusion Complex

**DOI:** 10.3390/ijms252312778

**Published:** 2024-11-28

**Authors:** Federica De Gaetano, Loredana Leggio, Consuelo Celesti, Fabio Genovese, Marco Falcone, Salvatore Vincenzo Giofrè, Nunzio Iraci, Nunzio Iraci, Cinzia Anna Ventura

**Affiliations:** 1 Department of Chemical, Biological, Pharmaceutical and Environmental Sciences, University of Messina, Viale Ferdinando Stagno d’Alcontres 31, 98166 Messina, Italy; 2 Department of Biomedical and Biotechnological Sciences (BIOMETEC), University of Catania, Torre Biologica, Via Santa Sofia 97, 95125 Catania, Italy; loredana.leggio@unict.it (L.L.); nunzio.iraci@unict.it (N.I.); 3 Department of Engineering, University of Messina, Contrada Di Dio, 98166 Messina, Italy; ccelesti@unime.it

**Keywords:** cinnamic acid, randomly methylated β-cyclodextrin, inclusion complex, physicochemical characterization, biological in vitro study

## Abstract

Cinnamic acid (CA) has many beneficial effects on human health. However, its poor water solubility (0.23 g/L, at 25 °C) is responsible for its poor bioavailability. This drawback prevents its clinical use. To overcome the solubility limits of this extraordinary natural compound, in this study, we developed a highly water-soluble inclusion complex of CA with randomly methylated-β-cyclodextrin (RAMEB). The *host-guest* interaction was explored in liquid and solid states by UV-Vis titration, phase solubility analysis, FT-IR spectroscopy, and ^1^H-NMR. Additionally, molecular modeling studies were carried out. Both experimental and theoretical studies revealed a 1:1 CA/RAMEB inclusion complex, with a high apparent stability constant equal to 15,169.53 M^−1^. The inclusion complex increases the water solubility of CA by about 250-fold and dissolves within 5 min. Molecular modeling demonstrated that CA inserts its phenyl ring into the RAMEB cavity with its propyl-2-enoic acid tail leaning from the wide rim. Finally, a biological in vitro study of the inclusion complex, compared to the free components, was performed on the neuroblastoma SH-SY5Y cell line. None of them showed cytotoxic effects at the assayed concentrations. Of note, the pretreatment of SH-SY5Y cells with CA/RAMEB at 10, 30, and 125 µM doses significantly counteracted the effect of the neurotoxin MPP^+^, whilst CA and RAMEB alone did not show any neuroprotection. Overall, our data demonstrated that inclusion complexes overcome CA solubility problems, supporting their use for clinical applications.

## 1. Introduction

In the search for novel biologically active compounds, natural chemicals have a high potential for development into drugs [1,2]. Although many molecules from natural resources have promising pharmacological activity, only a few molecules are selected as candidates for drug development [3]. This is mainly due to the cost and lengthy process of initial screening, which involves numerous in vitro biological assays to confirm physiologically relevant activity and the absence of toxicity [4]. Furthermore, most of them have poor water solubility, which prevents their easy clinical application. Poor solubility in biological fluids drastically reduces their bioavailability.

**Figure 1:**
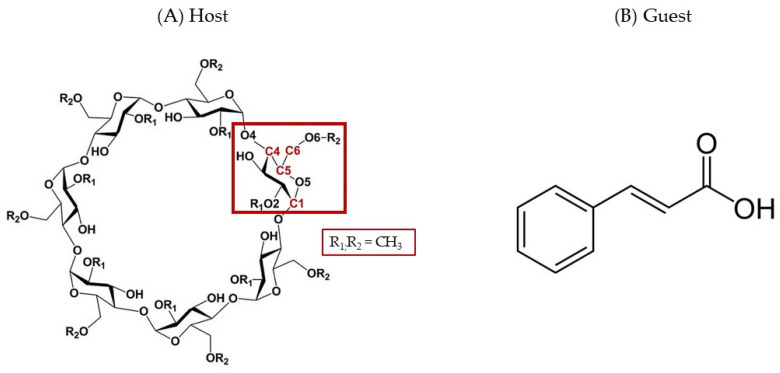
Schematic representation of (**A**) RAMEB (the positions of the carbon atoms with numerical indexes in each glucose unit are C1, C4, C5, and C6, as highlighted in the red box) and (**B**) CA.

Cinnamic acid (CA) (Figure 1B) is a natural plant compound, commonly in the trans-geometry, present in high concentrations in fruits, vegetables, cereals, coffee, tea, and wine [5,6,7]. The beneficial effect of CA for the prevention and management of diabetes is largely known [8,9]. Oral administration of CA prevented the development of diabetic cardiomyopathy due to its cardioprotective, anti-dyslipidemia potential anti-inflammatory, and antidiabetic properties [9]. CA has attracted much attention due to its anticancer, antioxidant, antimalarial, and antimicrobial properties. Liu et al. discovered that in human melanoma cells, CA enhanced tumor cell differentiation by modifying the expression of genes linked to tumor metastasis and immunogenicity [10]. Through the activation of caspase 9, CA effectively reduced the viability of HT-144 melanoma cells [11]. The bioactive molecule was shown to act as an effective anticancer agent, inducing apoptosis in breast cancer cells via TNFA-TNFR1-mediated extrinsic apoptotic pathway [12]. CA antioxidant activity has been widely discussed and demonstrated by several authors [13]. It is able to clear oxygen-free radicals in the body, improving immunity and delaying human aging [14]. Recently, an interesting work [7] demonstrated that CA effectively halts the progression of Sandhoff disease by a PPARα-dependent mechanism, increasing the longevity of Sandhoff mice. Similarly, other authors demonstrated involvement of PPARα by CA, showing neuroprotective effects on a model of Parkinson’s disease (PD) [15]. Thus, CA can be considered a promising starting compound for the development of new, highly effective drugs [16,17]. Despite these interesting properties, the biggest problem of CA is the poor water solubility (0.23 g/L at 25 °C) [18], which in vivo causes poor bioavailability at the site of action [19], so limiting its clinical application.

Preparation of inclusion complexes with cyclodextrins (CDs) represents an excellent way to overcome solubility problems of synthetic drugs [20,21,22] or natural products [23,24,25,26]. They are cyclic oligosaccharides derived from starch through enzymatic hydrolysis, which possess a unique truncated cone structure with an outer hydrophilic layer and an inner surface with lipophilicity comparable to an aqueous ethanolic solution [27]. The internal cavity hosts lipophilic molecules, forming *host-guest* interactions, which allows for application advantages such as increased solubility and chemical stability and delivery of the included bioactive compounds derived from natural sources [28]. Generally, CDs have a low toxicity profile and have been approved by the Food and Drug Administration (FDA) for administration in animals and humans [29]. Their application in the pharmaceutical field has been of great relevance, developing innovative drug delivery systems [27,30]. They greatly increase the solubility of drugs in hydrophilic environments, allowing the development of liquid pharmaceutical forms [31]. They protect the included molecules against environmental impacts, such as heat and oxidation [32,33], and can provide their sustained release [34,35], also improving cellular uptake and absorption [36]. Furthermore, they can be used in combination with other drug delivery systems to improve their technological properties [37,38,39,40], or themselves show the ability to form nanoparticles [41,42]. CDs are frequently utilized to mask taste for pharmaceutical purposes. This trait has multiple explanations. First, unlike free medicines, complexes may not exhibit affinity for taste receptors because of apparent structural differences. Smaller doses of medications, below the bitterness threshold, may also be present in the mouth as a result of the gradual release [43,44]. The most common native CDs have 6, 7, or 8 glucose units, namely α-, β-, and γ-CD, and although they are hydrophilic, their solubility in water is relatively low due to their tendency to aggregate by forming intermolecular hydrogen bonds [45]. This is why they have been functionalized with different alkyl chains or other groups to significantly increase solubility in water and biological fluids [46]. The most important chemical modifications of CDs for their application in food, agriculture, industry, pharmaceutical formulations, and biomedical applications [47] concern the insertion of hydroxypropyl or sulfobutyl ether chains or methoxyl groups into the β-CD structure. The resulting macrocycles show excellent water solubility and complexation properties together with a very low toxic profile. Hydroxypropyl-β-CD (HP-β-CD) and sulfobutylether-β-CD (SBE-β-CD) are approved by the FDA for parenteral administration, and randomly methylated β-CD (RAMEB) (Figure 1A) is today present in the market within products for ophthalmic and nasal administration.

There are several articles in the literature regarding the synthesis of the inclusion complex of CA with native CDs. Specifically, the inclusion into α-CD improves the antimicrobial activity of the bioactive molecule against *Escherichia coli* and *Salmonella enterica* suspended in apple cider and orange juice, respectively [48]. In a study of CA complexation with native CDs, authors [49] found that all cases resulted in the formation of a 1:1 inclusion complex; however, the stronger host-guest interaction was obtained for the CA/β-CD inclusion complex. Recently, a paper was published concerning the complexation of CA and its *trans*-derivatives into β-CD and HP-β-CD. The authors adopted a conductimetric method to characterize the inclusion complexes, demonstrating a 1:1 stoichiometry for all derivatives and enhanced stability at decreased macrocycle molar mass [50]. No biological studies were performed.

To the best of our knowledge, there are no papers in the literature concerning the host-guest interaction between CA and RAMEB, nor the influence of this macrocycle on the water solubility and neuroprotective effect of CA.

Within this framework, the goal of this study was to overcome the solubility limits of CA by developing a highly water-soluble inclusion complex with RAMEB (CA/RAMEB). RAMEB exhibits excellent ability to improve water solubility and stability of lipophilic molecules and their permeation through the viable membranes [32,51]. It can reversibly alter the membrane permeability by extracting cholesterol and phospholipids and complexing them in the aqueous layer [52,53]. Furthermore, Fenyvesi et al. [49,54] demonstrated that labeled RAMEB is able to enter epithelial cells by fluid-phase endocytosis, contributing to the enhancement of paclitaxel oral bioavailability. RAMEB is considered a safe candidate as an excipient for drug delivery. Boulmedarat et al. conducted a study on the toxicity of RAMEB on buccal mucosa using a reconstituted human oral epithelium model consisting of TR 146 cells. Concentrations between 2% and 5% (*w*/*v*) did not induce tissue damage even after 5 days of repeated exposures. Whereas the higher dose of 10% causes cytotoxic and inflammatory effects depending on the exposure time [55].

Furthermore, RAMEB at 10% (*w*/*v*) did not cause nasal membrane damage after up to 1 h exposure [56].

Based on this evidence, maintaining concentrations of less than 5% (*w*/*v*) RAMEB could enhance the pharmacological activity of CA without damaging cells.

The *host-guest* interaction was investigated by several techniques, in solution and solid state. The experimental results were confirmed by molecular modeling studies. Finally, biological in vitro studies on the neuroblastoma SH-SY5Y cell line were carried out to evaluate the tolerability and neuroprotective effect of the CA/RAMEB inclusion complex against the PD neurotoxin 1-methyl-4-phenylpyridinium (MPP^+^).

## 2. Results and Discussion

### 2.1. In-Solution Study on the Host-Guest Interaction of the CA/RAMEB Inclusion Complex

**Figure 2:**
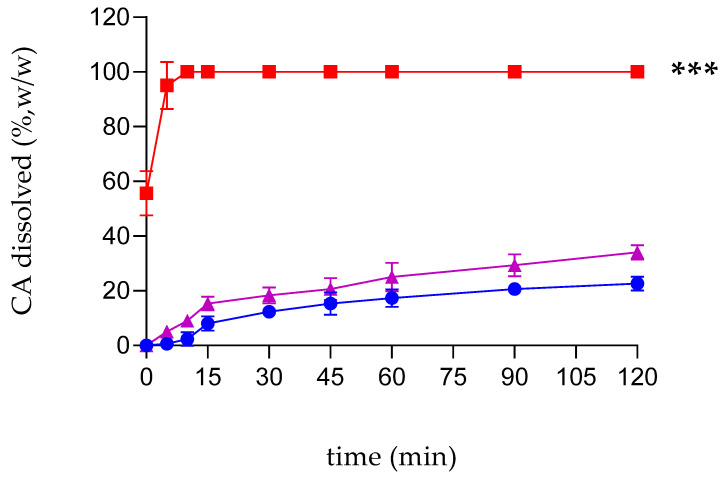
Dissolution profiles of free CA (circles and blue line) as compared to the physical mixture (triangles and purple line) and the inclusion complex (squares and red line) in water at 25 ± 0.5 °C. The experiments were carried out in triplicate. The results are presented as the mean of three different experiments ± standard deviation (SD). The error bar, if not shown, is inside the symbol. CA/RAMEB inclusion complex values are statistically significant compared to free CA data (*** *p* < 0.001).

The poor water solubility of a new pharmacological compound represents a barrier to its introduction into the market [57]. The complexation of CA within RAMEB produced a white powder with high water solubility. An increase of about 250-fold in the CA solubility was in fact detected (0.23 g/L to 58.5 g/L for free CA and complexed CA, respectively). Because of the improved solubility, a fast dissolution of the complex was observed. As shown in Figure 2, about 55% (*w*/*w*) of the CA/RAMEB inclusion complex was immediately dissolved, and within 10 min dissolution was quantitative. This trend is also due to the high wettability and reduced crystallinity of the freeze-dried complex. In contrast, the dissolution profiles of free CA and the CA/RAMEB physical mixture are almost overlapping. At the end of the experiment, the free CA dissolves to about 22% (*w*/*w*) while the physical mixture is 35% (*w*/*w*).

**Figure 3:**
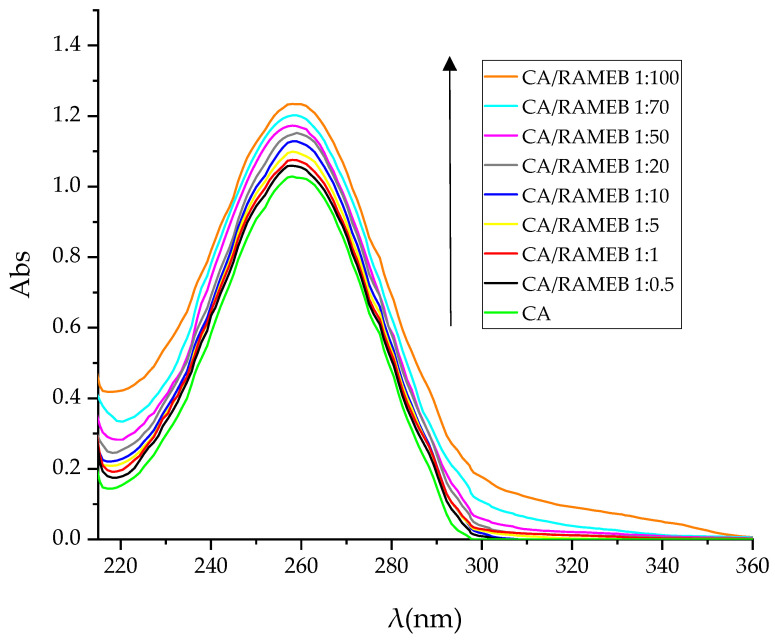
UV-vis spectra of free CA and in the presence of an increasing amount of RAMEB in water at 25.0 ± 0.5 °C. The experiments were carried out in triplicate.

The complexation was studied by UV-vis titration. The spectra of free CA and with increasing concentrations of RAMEB are shown in Figure 3.

CA exhibits its characteristic absorption peak at 268 nm due to the π–π∗ transition. At the increase in the macrocycle concentration, the absorbance of the CA characteristic peak progressively increases. This suggests a transfer of CA from a polar environment to a non-polar microenvironment and the establishment of an interaction between the phenyl ring of CA and the CD cavity.

**Figure 4:**
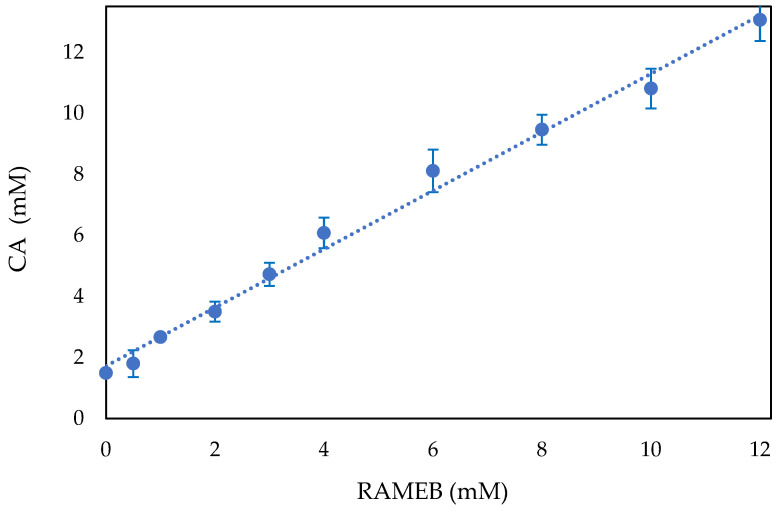
Phase-solubility diagrams of CA/RAMEB at 25.0 ± 0.5 °C. The experiments were carried out in triplicate ± standard deviation (SD). The error bar, if not shown, is inside the symbol.

The host-guest complexation was confirmed by phase solubility studies conducted in water, which also supports the NMR results and modelling study below in that a 1:1 complex was formed. The impact of CD on the physicochemical characteristics of the guest molecules may be reflected in the stability or equilibrium constants of the guest–CD complexes. The phase solubility approach, which was the most helpful analytical technique for the complex, was employed in this work to obtain the apparent stability constant (Kc), as reported by Higuchi and Connors. The phase solubility diagram (Figure 4) displayed a typical AL curve, in which the water solubility of CA increased linearly with increasing RAMEB concentration, which could form a 1:1 stoichiometric ratio inclusion complex.

Using the slope and y-intercept from the equation of the line (R^2^ = 0.9917 ± 0.0174) of the CA/RAMEB phase solubility diagram, the calculated Kc was 15,169.53 M^−1^ ± 0.1888 M^−1^. This high value can be attributed to a strong host-guest interaction of the CA with both the CD cavity and the external methyl groups, which stabilize the complexation.

Complexation efficiency (CE), which was determined using the slope of the AL-type isotherm (Equation (2), is equal to 22.63. The calculated CA:RAMEB molar ratio was 1:1, according to Equation (3). Finally, following Equation (4), the solubilization coefficient (SC) was calculated to be equal to 8.66.

These results confirm that RAMEB has a good solubilization effect on CA.

### 2.2. In Solid-State Study by Fourier Transform Infrared (FT-IR) and Thermogravimetric Analysis (TGA)

**Figure 5:**
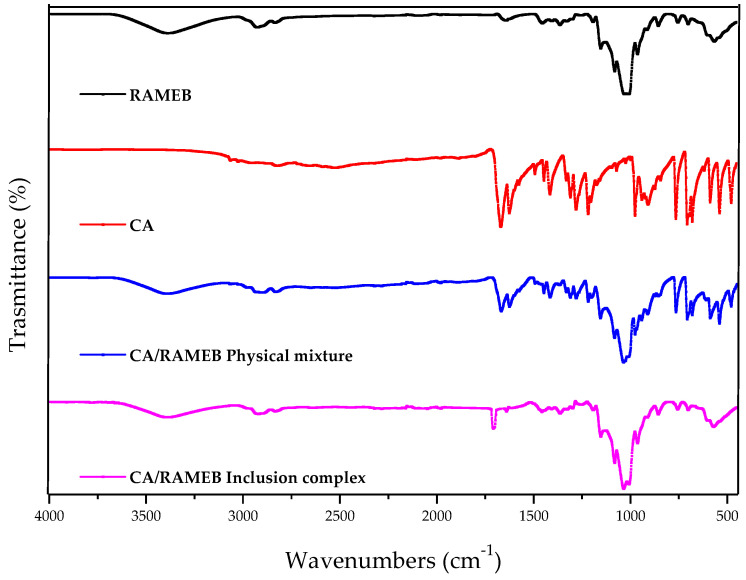
FT-IR spectra of the CA/RAMEB inclusion complex compared to the free components and the physical mixture.

The *host-guest* interaction in the solid state was studied by FT-IR (Figure 5). The infrared spectrum of CA shows in the region at ~3400 and ~2300 cm^−1^ two broad overlapping bands of O-H and C-H stretching vibrations. The bands are complicated by the hydrogen bonding that affects the frequencies of the O-H vibrations (as part of the carboxylic acid functional group), and the C-H vibrations vary due to the different origins, i.e., the C-H vibrations of the alkene from the CH=CH group and the C-H vibrations of the arene from the benzene ring. The peak at ~1680 cm^−1^ is due to the C=O stretching vibrations of the carbonyl group (of the carboxylic acid functional group). The C=O absorption is clearly distinguished from the C=C stretching band of the alkyne, which peaks at ~1630 cm^−1^. Furthermore, in the range from 1500 to 400 cm^−1,^ we find the fingerprint region for the identification of CA and most organic compounds. RAMEB shows a broad absorption band in the 3600–3100 cm^−1^ region corresponding to -OH stretching vibration from unmethylated hydroxyl moieties, and a broad region below 1500 cm^−1^ that has distinct peaks, which is most likely characteristic of the cyclodextrin ring. FTIR spectroscopy for the physical mixture showed the presence of the main characteristic peaks of both RAMEB and CA, although with a decrease in the intensity of the characteristic bands of CA. The FTIR spectrum of the inclusion system showed a marked decrease in the intensity of the characteristic bands of CA and a shift to several wavenumbers, attributable to the formation of stable hydrogen bonds in the inclusion complex. These data demonstrate the stable interaction between CA and RAMEB.

**Figure 6:**
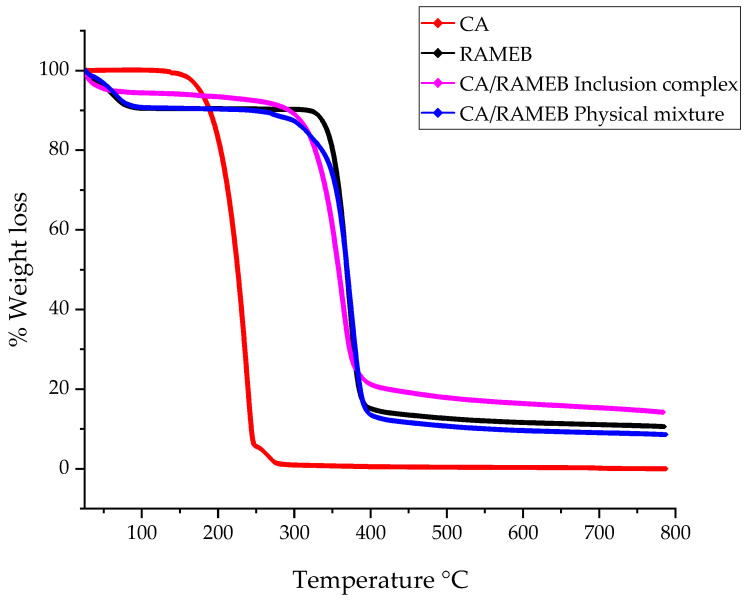
TGA thermograms of the CA/RAMEB inclusion complex compared to the free components and the physical mixture.

The lyophilized CA/RAMEB inclusion complex was subjected to thermogravimetric analysis (TGA) and compared with free CA, RAMEB, and the physical CA/RAMEB mixture. Figure 6 shows the TG curves obtained.

The physical mixture of CA/RAMEB and RAMEB shows a slight comparable initial weight loss up to 90 °C, probably caused by evaporation of absorbed water on the macrocycle surface [58,59]. The degradation of free CA exclusively presented the weight loss in the range of 120–270 °C due to its degradation and leaves no residue. The TG curve of RAMEB presented significant weight loss in the range of 320–400 °C, leaving a residue of about 10%. The TG curve of the physical CA/RAMEB mixture was similar to that of free RAMEB, but degradation starts at lower temperatures, between 250 and 400 °C, leaving a residue of about 8.5%. A different trend was observed for the inclusion complex. Initial water loss is also present, but it is greatly reduced up to 50 °C. Thereafter, degradation of the complex is initiated at a slightly lower temperature than that of the free RAMEB, between 220 and 400 °C. A residue of about 15 percent, higher than that of the physical mixture and free components, is present, probably indicating an increase in the thermal stability of the CA, produced by the complexation process.

The shelf life of the solid inclusion complex was evaluated over time in different temperature conditions (25 °C, 37 °C, and 50 °C). During this time the complex was subjected to TGA, obtaining no difference in the TG curves within the entire experimental time. This demonstrated high stability of CA/RAMEB inclusion complex. As an example, Figure S7 shows the TGA carried out after two months of storage at 50 °C.

### 2.3. Molecular Modeling Studies

Molecular mechanics simulations were employed to gain insight into the CA/RAMEB interaction. Briefly, CA was docked into RAMEB by the use of replica exchange with solute-tempering molecular dynamics simulations (see the experimental section). Cluster analysis of the replica 0 trajectory (T = 300 K) revealed the presence of just one significantly (population > 50/4000) populated cluster (population 2263/4000).

**Figure 7:**
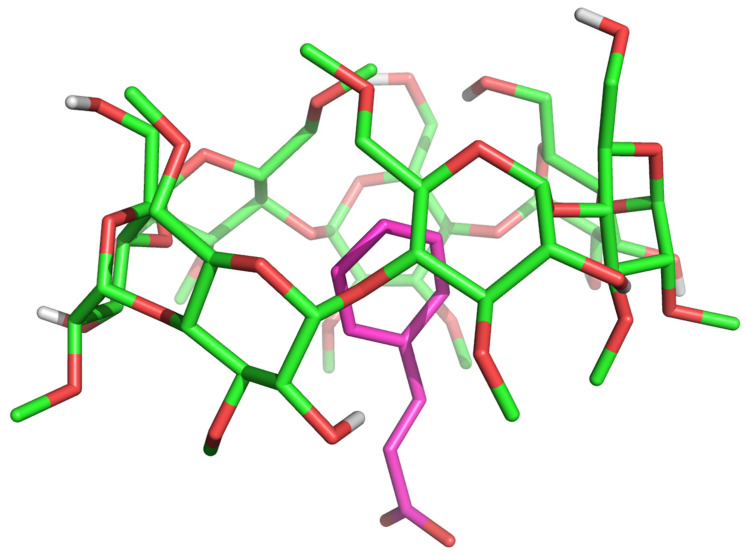
Representative conformation of the most populated CA/RAMEB complex cluster from REST MD simulation (replica 0–300 K). CA is represented in magenta sticks whilst RAMEB is depicted in green sticks.

**Figure 8:**
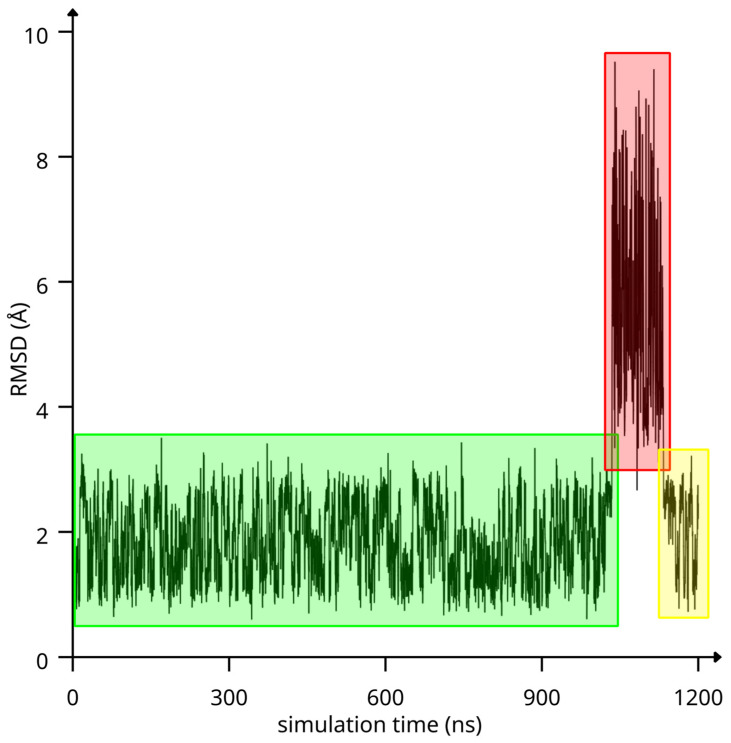
RMSD of CA and RAMEB heavy atoms as a function of simulation time. Low RMSD simulation time windows are highlighted in green and yellow, while the red area highlights the high RMSD simulation time window.

In this bound conformation (Figure 7), CA inserts its phenyl ring into the RAMEB cavity with its propyl-2-enoic acid tail leaning from the wide rim, where it is not found to specifically interact with any group in position 2 or 3 of RAMEB. This CA/RAMEB conformation was then submitted to a 1.2 µs-long MD simulation to investigate the energetics that mostly contribute to complex stability. The complex stood stable until 1034 ns of simulation time (Figure 8—green area), after which it dissociated (Figure 8—red area), with CA wandering in the water buffer and associating again with RAMEB at around 1133 ns of simulation time (Figure 8—yellow area).

**Figure 9:**
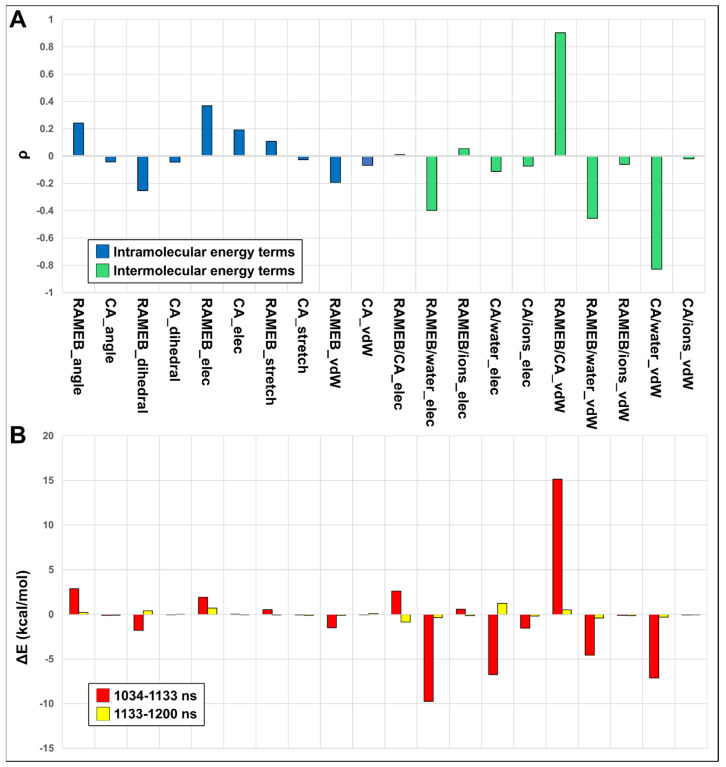
(**A**) Pearson correlation values (ρ) of RMSD with intra- (blue bars) and inter- (green bars) molecular VdW and electrostatic energy terms. (**B**) Variations of electrostatic and VdW energies terms between the 0 and 1034 ns simulation time window (set to zero); the 1034 and 1133 ns (red bars); and the 1133 and 1200 ns (yellow bars) simulation time windows.

The spontaneous dissociation and association observed during the MD simulation enabled us to correlate the binding/unbinding motions to the energetics recorded during the simulation (Figure 9A).

It clearly appears that the complex is mainly stabilized by intermolecular Van der Waals (VdW) interactions between CA and RAMEB (Figure 9). Indeed, considering that complex dissociation relates to high RMSD values, it is evident, from the RMSD/energy term correlation values, that when CA unbinds from RAMEB, RAMEB/WATER electrostatics and RAMEB/WATER and CA/WATER VdW interaction energies become more favorable, but this energy gain is coupled to a significant loss of favorable RAMEB/CA VdW energy (Figure 9A). This can be highlighted even in the energy differences found between the 0 and 1034 ns; 1034 and 1133 ns; and 1133 and 1200 ns simulation time windows (Figure 9B), where ΔE values show that in the 1034–1133 ns time window, when CA and RAMEB are dissociated (1034–1133 ns—red bars in Figure 9B), both electrostatic and VdW interaction energies of CA and RAMEB with the solvent become more favorable to the detriment of CA/RAMEB VdW and electrostatic energy. When the complex is instead associated again in the 1133–1200 ns time window (yellow bars in Figure 9B), the E values tend to revert back to the values observed in the 0–1034 ns time window.

### 2.4. Nuclear Magnetic Resonance Measurements

**Figure 10:**
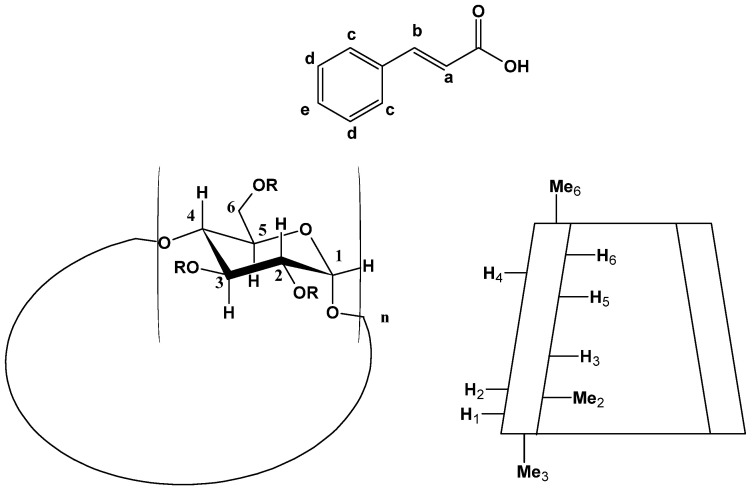
Structure of CA and general structure of RAMEB (n = 7).

To confirm the clues we obtained from in silico studies, we conducted further experimental studies by means of nuclear magnetic resonance (NMR). One of the most suitable methods for the quantification of non-covalent molecular interactions is ^1^H-NMR spectroscopy, which also enables us to obtain information about inter- and intramolecular interactions between atoms of the host and guest molecules [60,61,62]. At the same time, the observed chemical shift changes (Δδ) provide insight into the structure of the complexes. To better understand the interactions between the guest and the host, the structure of the CA and the schematic representation of the RAMEB are shown in Figure 10.

**Figure 11:**
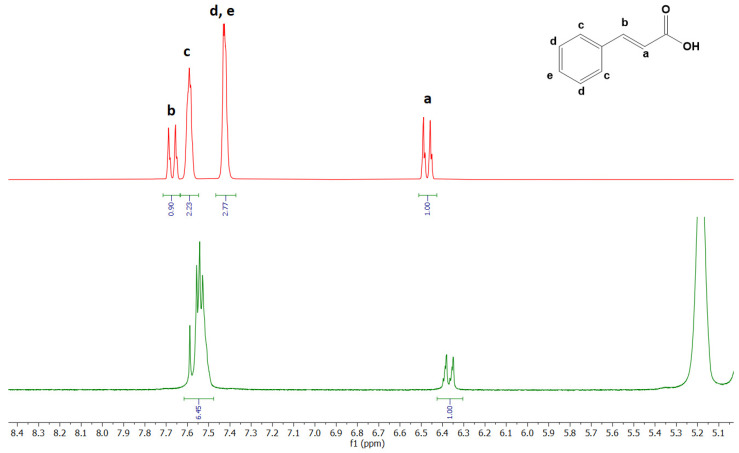
Stacked portions of ^1^H-NMR spectra relative to free CA (red spectra) and CA/RAMEB inclusion complex (green spectra). Full spectra are reported in Figures S1–S3.

During the complexation of CA with RAMEB, the chemical and electronic environments of the nucleus are affected; therefore, the chemical shift of protons of CA is diagnostic. This was confirmed by changes in the chemical shift of CA protons relative to the protons of the free molecule (see Figure 11, Table 1, and Figure S1). In the CA/RAMEB inclusion complex, the chemical shift of olefin protons H-a decreases from 6.49 to 6.37 ppm with the upfield shift of Δδ 0.12 ppm. Similarly, olefin protons H-b decrease from 7.69 to 7.59 ppm with the upfield shift of Δδ 0.1. Furthermore, the aromatic protons show in the spectrum of the free molecule a chemical shift of 7.59 ppm (ortho) and 7.43 ppm (meta and para), while in the inclusion complex, they appear as a multiplet at 7.54 ppm with the upfield shift of Δδ 0.05 ppm and with the downfield of Δδ −0.11 ppm for ortho and meta/para protons, respectively.

**Figure 12:**
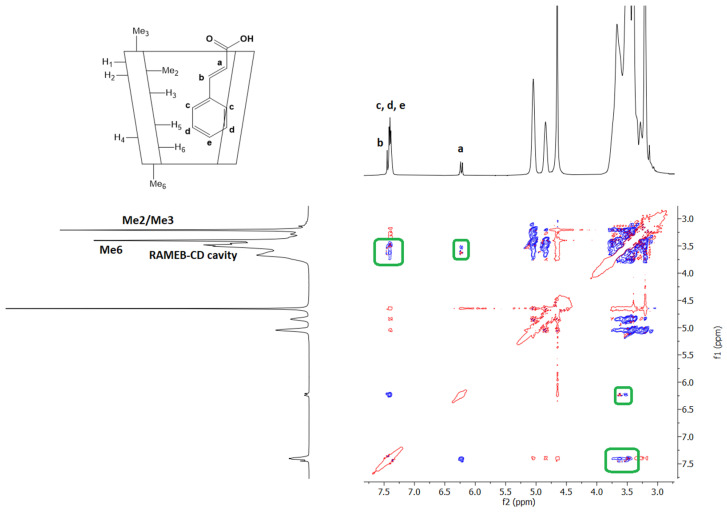
Expansion of two dimensional ROESY-AD plot of the CA/RAMEB complex in D_2_O/CD_3_OD (80:20 *v*/*v*). Blue and red colour represented negative- and positive-phase signals, respectively. Only the cross-peaks of interest were highlighted with green squares.

The postulated complexation of CA with RAMEB was also demonstrated via NMR spectroscopy using rotating frame Overhauser effect spectroscopy (ROESY) experiments, where if two protons from different compounds are in the spatial vicinity within 3–5 Å, an ROE cross-peak is observed in the 2D ROESY spectrum (Figure 12 and Figure S4). The ROE cross-peaks between the olefin protons H-a, H-b, and aromatic protons of the CA with the RAMEB internal cavity confirm the complexation. Furthermore, the absence of ROE cross-peaks between olefin and CA aromatic protons with Me_3_ of RAMEB confirms the in silico-predicted orientation of CA into the inclusion complex.

### 2.5. In Vitro Studies on Neuronal SH-SY5Y Cells

**Figure 13:**
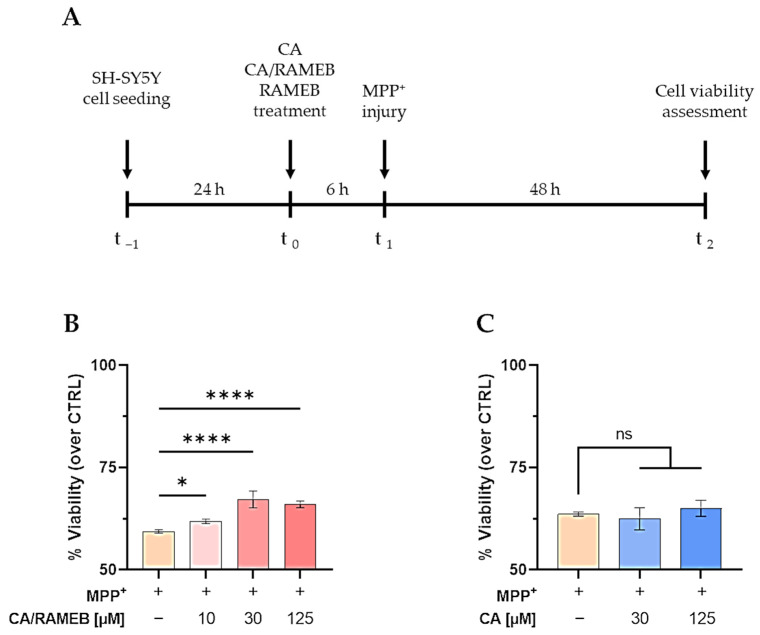
CA-RAMEB complex is neuroprotective against MPP^+^ neurotoxin. (**A**) Schematic representation of the experimental protocol. (**B**,**C**) Analysis of the neuroprotective effects of CA/RAMEB (**B**) and CA alone (**C**) under 2 mM MPP^+^ treatment for 48 h. Data are presented as mean ± SD of three independent experiments. One-way ANOVA with Dunnett’s multiple comparisons versus MPP^+^: * *p* < 0.05, **** *p* < 0.0001, ns: not significant.

The effects of CA, CA/RAMEB, and RAMEB were evaluated on the neuroblastoma SH-SY5Y cell line. First, cell viability was measured in the presence of increasing concentrations of the compounds upon 48 h of incubation (Figure S5A). The results showed that all concentrations of CA, CA/RAMEB complex, and RAMEB did not show cytotoxicity on SH-SY5Y cells, confirming their suitability for neuroprotective studies (Figure S5A). To assess the protective potential of the CA/RAMEB complex over CA and RAMEB alone, cells were exposed for 6 h to the compounds, and viability was evaluated under 48 h treatment with 2 mM 1-methyl-4-phenylpyridinium (MPP^+^), a neurotoxin used in vitro to mimic PD (Figure 13A). MPP^+^ affects mitochondrial functionality by inhibiting the activity of NADH-ubiquinone oxidoreductase (complex I), resulting in neuronal impairment/death. The treatment with MPP^+^ induced a 40% reduction in cell viability compared to untreated neuronal cells at 48 h (Figure 13B). Notably, the pretreatment with 10, 30, and 125 µM CA/RAMEB complex significantly counteracted MPP^+^-induced toxicity (Figure 13B). At the same concentrations, CA and RAMEB alone did not have any effect on MPP^+^-injured SH-SY5Y cells, thus supporting the efficiency of the RAMEB complex system for the delivery and the neuroprotective function of CA (Figure 13C and Figure S5B).

## 3. Materials and Methods

### 3.1. Materials

Cinnamic acid (CA, MW 148.16 g/mol, C_9_H_8_O_2_) and all analytical grade reagents were purchased from Sigma-Aldrich (St. Louis, MO, USA). Randomly methylated β-cyclodextrin (RAMEB, average degree of substitution 11.2, MW 1310 g/mol) was kindly provided by Cyclolab R&D. Ltd. (Budapest, Hungary). Water used throughout this study was double distilled, then filtered through 0.22 µm Millipore^®^ GSWP filters (Bedford, MA, USA).

### 3.2. Preparation of the Inclusion Complexes and Physical Mixture

The CA/RAMEB inclusion complex in a 1:1 molar ratio was prepared following the lyophilization method. RAMEB (175.5 mg, 6.75 M^−3^) was solubilized in 10 mL of water, then CA (10 mg, 6.75 M^−3^) was added, and the suspension was mechanically stirred for 1 h at 600 rpm. At the end of stirring, a solution was obtained, which was lyophilized (freeze dryer Alpha 1–4 LSCbasic, Martin Christ Gefriertrocknungsanlagen GmbH, Osterode/Harz, Niedersachsen, DE, Germany) for 48 h.

By carefully combining a precisely weighed quantity of drug and RAMEB in a mortar until the mixture was uniformly colored, a 1:2 molar ratio CA/RAMEB physical mixture was produced.

### 3.3. Solubility and Dissolution Profile in Water

The water solubility of the CA/RAMEB inclusion complex was assayed in the absence of light at 25 ± 0.5 °C. An excess amount of the inclusion complex was dispersed into water (10 mL) and maintained under magnetic stirring until the equilibrium was reached. At timed intervals, an aliquot of sample was withdrawn, filtered (Sartorius Minisart-SRP 15-PTFE filters with a pore size of 0.22 µm, Bedford, MA, USA), and the solution was analyzed by UV-vis spectroscopy for CA quantification. The equilibrium was reached in 24 h. The collected volume was replaced with fresh water, and the dates were corrected for dilution. The assay was carried out in triplicate.

In vitro dissolution profiles were obtained under low magnetic stirring (100 rpm) at 25 ± 0.5 °C by the 44th United States Pharmacopoeia (USP). Briefly, free CA (100 mg), IDE/RAMEB inclusion complex, and the physical mixture, both containing 100 mg of CA, were suspended separately in 900 mL of water. At fixed times, 5 mL aliquots of the dissolution medium were collected and filtered (Sartorius Minisart-SRP 15-PTFE filters with a pore size of 0.22 µm, Bedford, MA, USA), and the solutions were analyzed by UV-Vis spectroscopy to determine the amount of dissolved CA. The volume of the dissolution medium was kept constant throughout the experiment by adding fresh water to maintain sink conditions [63]. UV-vis apparatus and conditions were the same as described in the 2.2 paragraph. Three duplicates of each experiment were run, and the dilutions were used to adjust the data.

### 3.4. UV–Vis Titration Studies

Free CA (0.05 mg; 0.067 M^−3^) and increasing concentrations of RAMEB (ranging from 0.067 M^−3^ to 6.7 M^−3^) were solubilized in water and stirred before the analysis for 1 h, in the dark, at 400 rpm and 25.0 ± 0.5 °C [36]. Solutions were analyzed in the spectral range of 200–400 nm.

### 3.5. Phase-Solubility Studies

The phase-solubility diagram was constructed by Higuchi and Connors’s methods [64]. An excess amount of CA, beyond its intrinsic solubility (0.23 g/L [18]), was added to aqueous solutions containing increasing amounts of RAMEB (0–12 mM). We obtained suspensions that were kept in the dark, under magnetic stirring, at 25.0 ± 0.5 °C within a thermostatic bath (Telesystem 15.40, Thermo Scientific, Waltham, MA, USA), equipped with a temperature control unit (Telemodul 40C, Thermo Scientific, Waltham, MA, USA). After 24 h, the suspensions were filtered (Sartorius Minisart-SRP 15-PTFE filters with a pore size of 0.22 µm, Bedford, USA) and CA was quantified by UV-vis spectroscopy by using a StellarNet BLACK-Comet, model C, diode array spectrophotometer (Carlson Circle Tampa, Keystone, FL, USA), in the range 200–600 nm. The calibration curve was constructed in the range of concentration 0.0002–0.015 g/L, obtaining an R^2^ equal to 0.9975. The experiments were carried out in triplicate.

The association constant (Kc) of the complex was calculated by the following equation (Equation (1)) [64]:

Kc = slope/[(1 − slope)S_0_] (1)

where S_0_ is the intrinsic water solubility of CA.

From the slope of the phase-solubility diagram, we calculated the complexation efficiency (CE), according to Equation (2):

CE = slope/(1 − slope) (2)

The CA:RAMEB molar ratio was calculated as follows (Equation (3))

CA:RAMEB = 1/(1 + 1/CE) (3)

The solubilization coefficient (SC) was calculated as the ratio between CA solubility at the highest RAMEB concentration vs. solubility of free CA (Equation (4))

SC = A/A_0_ (4)

### 3.6. Fourier-Transform Infrared (FT-IR)

Infrared spectra were obtained with a Fourier transform infrared (FT-IR) Spectrum Two FT-IR spectrometer (PerkinElmer Inc., Waltham, MA, USA) by the ATR method in the range of 4000–450 cm^−1^, using a resolution of 4 cm^−1^. The spectra of each sample were recorded at a rate of 10 scans/min, resulting in 638 different points; the resulting data were collected in transmittance values (%) and processed using Origin software, OriginPro 2021 9.8.0.200, Copyright © 1991–2020 OriginLab Corporation (Northampton, MA, USA). Each formulation was tested in triplicate [65].

### 3.7. Thermal Gravimetric Analysis (TGA)

Thermogravimetric analyses were performed by means of a Discovery SDT 650 (TA Instruments—Waters LLC, Milford, MA, USA) in the temperature range between 25–800 °C, under a nitrogen flow rate of 100.0 mL/min and a heating rate of 20.00 °C/min. A weighed amount of free CA, RAMEB, CA/RAMEB inclusion complex, and CA/RAMEB physical mixture (5–10 mg) were poured onto a ceramic plate and analysed. Each sample was scanned in triplicate [65,66].

### 3.8. Stability Test of the Solid Inclusion Complex

The shelf life of the solid CA/RAMEB inclusion complex was evaluated by TGA [67,68], using the same apparatus and conditions as before described (see Section 3.7). The inclusion complex was stored, in the light, at different temperatures (25 °C, 37 °C, and 50 °C) for two months. At timed intervals (15 days, 1 month, and 2 months), the inclusion complex was subjected to TGA.

### 3.9. Molecular Modeling Studies

The RAMEB model was built using Maestro [69] upon a β-cyclodextrin crystal structure (CCDC entry 762697) [70]. RAMEB was modelled, analogously to previously reported studies [71], by adding 4 methyl groups at O2 positions of 2, 3, 4, and 6 glucopyranose units, 5 methyl groups at O3 positions of 1, 2, 4, 5, and 7 glucopyranose units, and 3 methyl groups at O6 positions of 1, 5, and 7 glucopyranose units.

The CA 3D structure was downloaded from PubChem [72]. The MD-simulated environment was built and run using Desmond [73]. CA and RAMEB were randomly placed in a water periodic boundary conditions simulation box. Solvation was treated explicitly using the TIP3P mode [74], and OPLS 2005 [75] was used as a force field. After the simulated environment was built, it was relaxed as previously reported [76]. And, finally, it was submitted to the MD production state. To enhance the conformational sampling, MD simulations were run using the replica exchange with solute tempering (REST) method [77].

Two MD replicas were run at 300 K and 394.94 K, respectively, setting the CA molecule as a hot region. Simulations were run for 480 ns in the isothermal-isobaric NPT ensemble, with time steps set to 2 fs, 2 fs, and 6 fs for bonded, near, and far interactions, respectively. The trajectories’ recording interval was set to 120 ps. The snapshots from replica 0 (300 K) were clustered according to their RMSD values, using average linkage and a 2.5 Å cutoff as merge distance. Non-hydrogen CA and RAMEB were used as reference atoms for both superimposition and RMSD calculation. The representative structure (the nearest to the cluster centroid conformation) from the most populated (2263/4000) cluster (conformation I—in which the CA propyl-2-enoic acid tail leaned from the wide rim) was then selected as the most favorable inclusion complex conformation.

To challenge this result, an upside-down CA/RAMEB inclusion complex, in which the CA propyl-2-enoic acid tail leaned from the narrow rim (conformation II), was manually built and submitted to metadynamics simulations. The simulated environment was prepared analogously to the above-described REST MD simulation. Two collective variables were set: CV1—the distance between the CA carboxyl carbon atom and the center of mass of the RAMEB oxygen atoms at C6; CV2—the distance between the center of mass of the CA phenyl atoms and the center of mass of the C1-O-C4 pattern atoms of RAMEB. After system relaxation, a 240 ns-long metadynamics simulation was run; addition of potentials was started after 12 ns of simulation. For both CV1 and CV2, repulsive Gaussian potential width was set to 0.05 Å, height to 0.03 kcal/mol, and the addition time interval to 0.09 ps. The free energy surface obtained from the simulation is reported in Figure S6.

Conformation I inclusion complex was then submitted to a 1.2 μs-long classical MD simulation, set up and run analogously to the previously described REST MD, with no replicas, to investigate the complex stability.

Correlations between *RMSD* values and force-field terms (*E*) were calculated using the Pearson correlation coefficient as follows:

$$\rho \left(RMSD,E\right)=\frac{cov\left(RMSD,E\right)}{\sigma_{RMSD}\cdot \sigma_{E}}=\frac{\sum_{i=1}^{n}\left((RMSD_{i}-{µ}_{RMSD}\right)\cdot \left(E_{i}-{µ}_{E}\right))}{\sqrt{\sum_{i=1}^{n}{\left(RMSD_{i}-{µ}_{RMSD}\right)}^{2}}\cdot \sqrt{\sum_{i=1}^{n}{\left(E_{i}-{µ}_{E}\right)}^{2}}}$$

where

$${µ}_{RMSD=}\frac{1}{n}\;\cdot \overset{n}{\underset{i=1}{\sum }}RMSD_{i}\;\;\;\;\;\;\;\;{µ}_{E=}\frac{1}{n}\;\cdot \overset{n}{\underset{i=1}{\sum }}E_{i}$$

Prior to correlation estimation, data points were interpolated as follows:

$$RMSD_{i}=\left\{\frac{\sum_{n=1+i}^{10+i}RMSD_{n}}{10}\;\right\}\;\;\;E_{i}=\left\{\frac{\sum_{n=1+i}^{10+i}E_{n}}{10}\right\}$$

### 3.10. Nuclear Magnetic Resonance Measurements

Samples of equivalent concentrations (15 mM) of CA, RAMEB, and CA/RAMEB (1:2 ratio) inclusion complexes were prepared in a D_2_O/CD_3_OD (80:20, *v*/*v*) solution and transferred to 5 mm NMR tubes for spectrum acquisition; the deuterated solvents were purchased from Merck (Life Science S.r.l., Milano, Italy). All spectra were recorded at 300 K with a Varian Unity Inova 500 MHz (11.75 T) spectrometer. D_2_O (4.79 ppm) was used as an internal reference to avoid the addition of external ones that could interact with RAMEB. For each sample, 32–64 scans were accumulated using either the standard presaturation or the double pulsed field gradient spin–echo (dpfgse) sequence for water suppression.

Rotating frame Overhauser effect spectroscopy (ROESY) spectra were recorded using the ROESYAD sequence (transverse cross-relaxation experiment in a rotating frame with adiabatic mixing pulses) using a mixing time of 500 ms [78].

### 3.11. SH-SY5Y Culture and Treatment

SH-SY5Y cells were purchased from ICLC (Interlab Cell Line Collection, accession number ICLC HTL95013; obtained from European Collection of Authenticated Cell Cultures (ECACC)) and cultured as described in [41,79,80]. Briefly, 2 × 10^4^ cells were seeded in black 96-well plates and treated with the indicated concentrations of both CA and CA-RAMEB complex. To test RAMEB alone, the doses were doubled to reproduce the CA/RAMEB molar ratio of 1:2. Cell viability was evaluated with the Cell-Titer-Blue Cell Viability Assay (Promega, Corporation, Madison, WI, USA, G8080) after 48 h. For the neuroprotective studies, the cells were pretreated with the compounds for 6 h and then exposed to 2 mM MPP^+^ (Sigma Aldrich, St. Louis, MO, USA, D048) for 48 h.

### 3.12. Statistical Analysis

All values are expressed as mean ± standard deviation (SD), and each analysis was executed three times. The results were analyzed by one- and two-way analysis of variance (ANOVA) followed by a Bonferroni or Dunnett post hoc test for multiple comparisons. A value of *p* < 0.05 was considered significant.

## 4. Conclusions

In this study, the CA/RAMEB inclusion complex was developed and characterized in solution and in solid state. The *host-guest* interaction was proved by UV-vis titration studies and confirmed by NMR spectroscopy. Both ROESY experiments and molecular modeling demonstrated CA inserts its phenyl ring into the RAMEB cavity with its propyl-2-enoic acid tail leaning from the wide rim, forming a stable complex with an association constant of about 15,169.53 M^−1^, demonstrated by phase-solubility studies. The complexation significantly improved the water solubility of CA of about 250-fold and produced a complete dissolution within 10 min. Finally, the potential neuroprotective effect of CA/RAMEB was tested in SH-SY5Y cells, widely used as a model for neurodegenerative studies, including PD. To induce neurodegeneration, cells were injured with MPP^+^, a well-known neurotoxin that targets the mitochondrial complex I, which induced ~40% neuronal death compared to untreated neuronal cells. This study demonstrated that the pretreatment with 10, 30, and 125 µM CA/RAMEB inclusion complex significantly counteracted MPP^+^-induced toxicity, while no effect was shown at the same concentrations of free CA. Indeed, key pathways affected by our treatments need to be further elucidated. However, our results confirm the advantage of using RAMEB for CA delivery.

## Figures and Tables

**Table 1:** ^1^H-NMR chemical shift (ppm) assignments for free CA and the CA/RAMEB inclusion complex.

Proton	CA	CA/RAMEB	Δδ *
a	6.49	6.37	+0.12
b	7.69	7.59	+0.1
c	7.59	7.54	+0.05
d	7.43	7.54	−0.11
e	7.43	7.54	−0.11

* Δδ = δ_CA_ − δ_CA/RAMEB_.

## Data Availability

The original contributions presented in the study are included in the article/Supplementary Materials, further inquiries can be directed to the corresponding author.

## Supplementary Materials

The following supporting information can be downloaded at: https://www.mdpi.com/article/10.3390/ijms252312778/s1.
