# Supplementary material for: Study of Host-Guest Interaction and In Vitro Neuroprotective Potential of Cinnamic Acid/Randomly Methylated β-Cyclodextrin Inclusion Complex
Source: Int J Mol Sci. 2024 Nov 28;25(23):12778. doi: 10.3390/ijms252312778 (PMC11641672; doi:10.3390/ijms252312778)
Supplement: Supplementary file 1 [file ijms-25-12778-s001.zip › ijms-3240355-supplementary.pdf]

International Journal of
Molecular Sciences

MDPI

Supplementary Materials

Table of content

- **Figure S1.** ^{1}H NMR spectrum of CA.

- **Figure S2.** ^{1}H NMR spectrum of RAMEB.

- **Figure S3.** ^{1}H NMR spectrum of CA/RAMEB.

- **Figure S4.** 2D ROESY-AD plot of CA/RAMEB (1:2 ratio) inclusion complex.

- **Figure S5.** (A) Analysis of cell viability of neuronal SH-SY5Y cells treated with increasing concentrations of CA, CA/RAMEB, and RAMEB. (B) Analysis of the potential neuroprotective effects of RAMEB under 2 mM MPP^{+} treatment for 48 hours.

-**Figure S6.** Free Energy Surface from CA/RAMEB metadynamics simulations.

- **Figure S7.** TGA thermograms of CA/RAMEB inclusion complex at 50°C for two months.

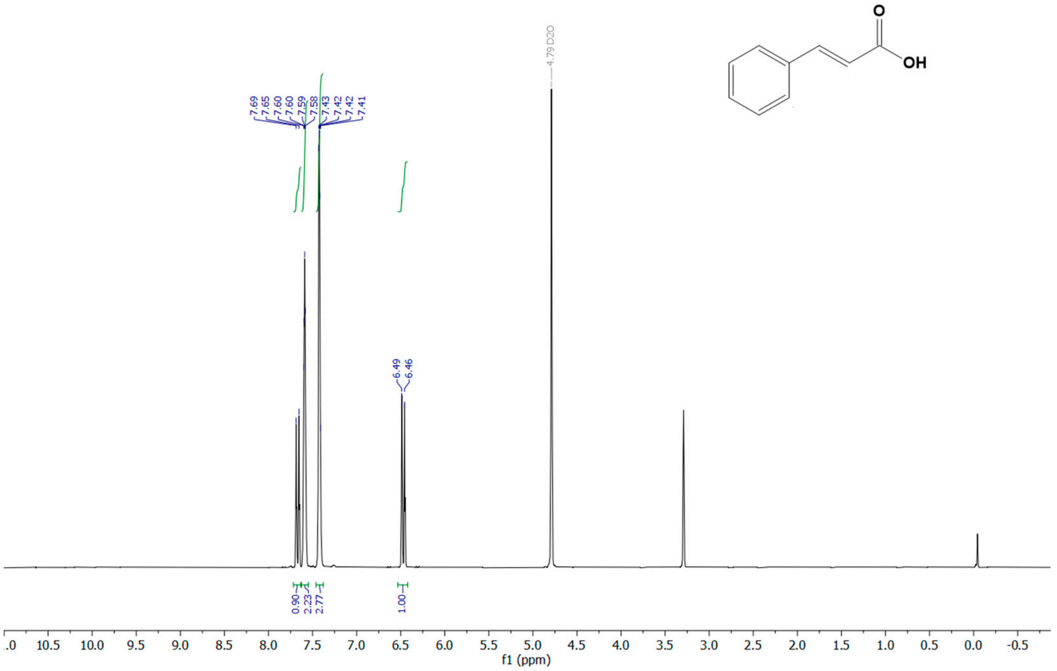

Figure S1. ^{1}H NMR spectrum of CA in D_2O/CD_3OD (80:20, v/v), recorded at 25 °C and 500 MHz.

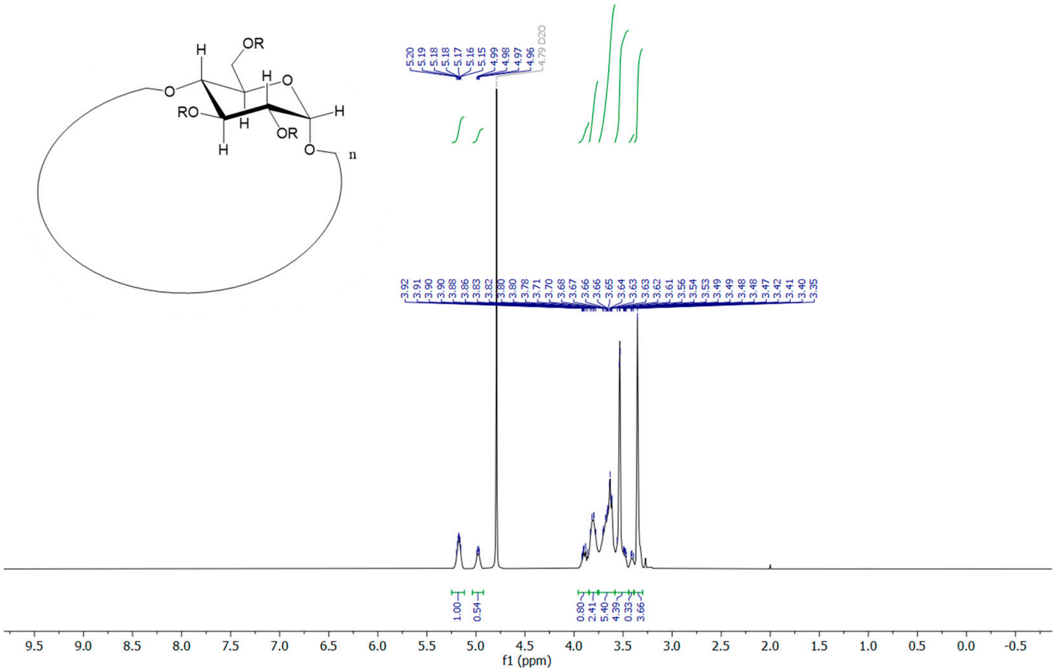

Figure S2. ^{1}H NMR spectrum of RAMEB in D_2O/CD_3OD (80:20, v/v), recorded at 25 °C and 500 MHz.

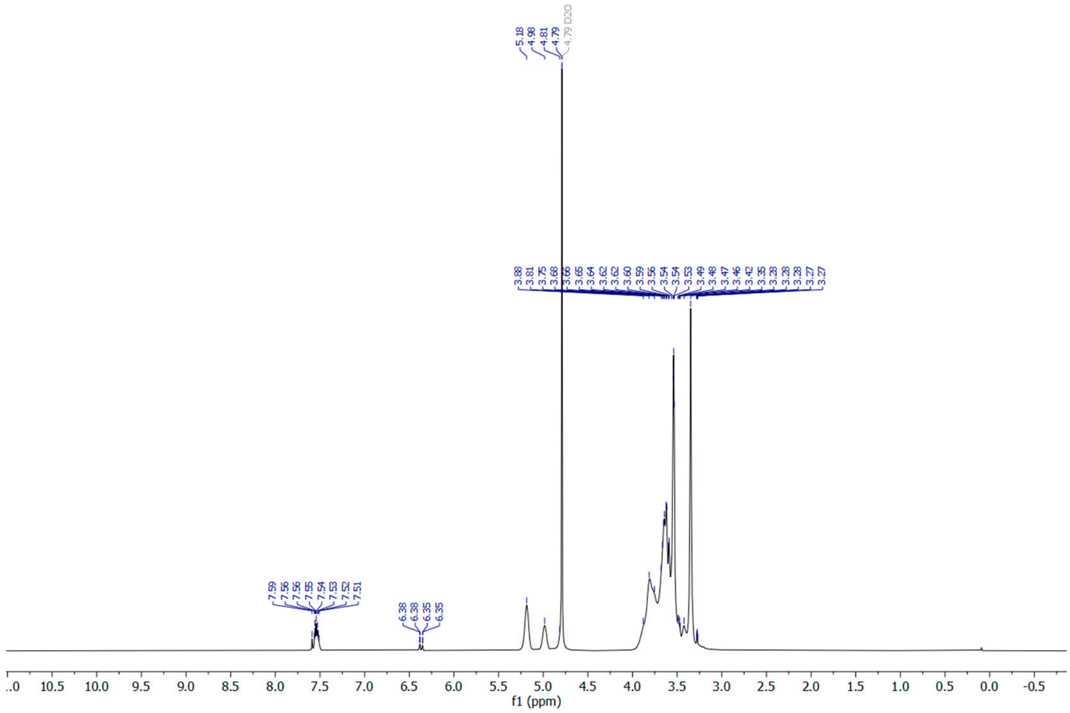

Figure S3. ^{1}H NMR spectrum of CA/RAMEB (1:2 ratio) inclusion complex in D_2O/CD_3OD (80:20, v/v), recorded at 25 °C and 500 MHz.

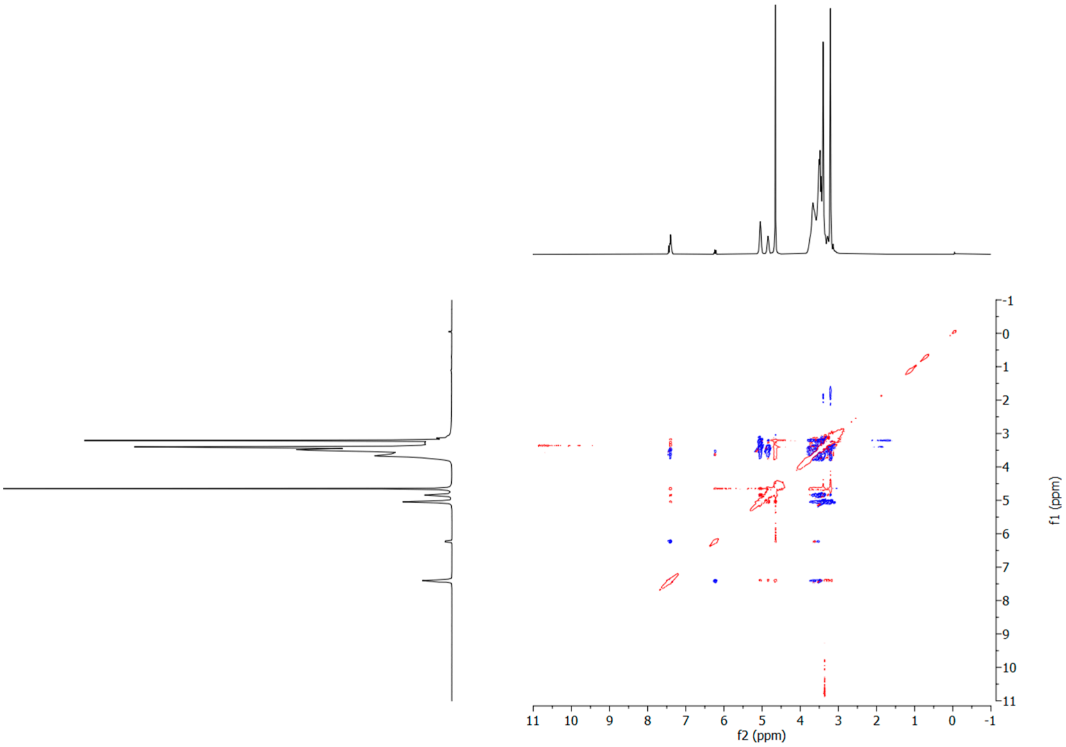

Figure S4. 2D ROESY-AD plot of CA/RAMEB (1:2 ratio) inclusion complex in D_2O/CD_3OD (80:20, v/v), recorded at 25 °C and 500 MHz.

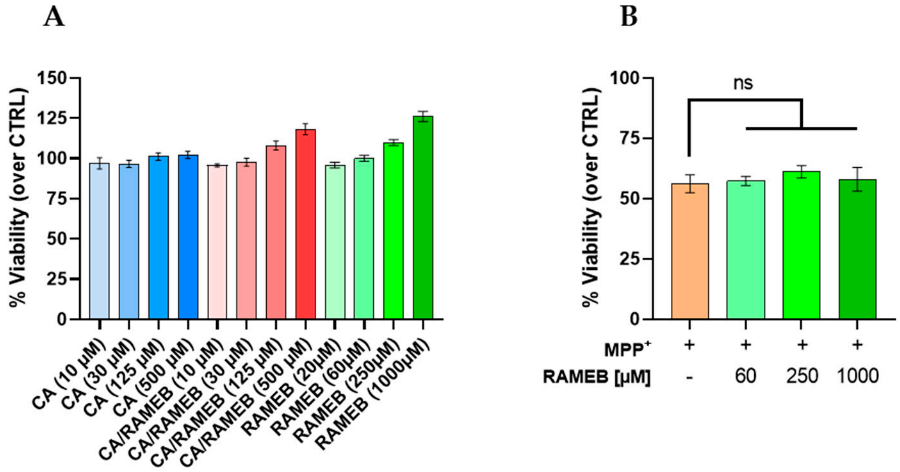

Figure S5. (A) Analysis of cell viability of neuronal SH-SY5Y cells treated with increasing concentrations of CA, CA/RAMEB, and RAMEB. (B) Analysis of the potential neuroprotective effects of RAMEB under 2 mM MPP^+ treatment for 48 hours. Data are presented as mean ± SD. One-way ANOVA with Dunnett's multiple comparisons versus MPP^+ in (B), ns: not significant.

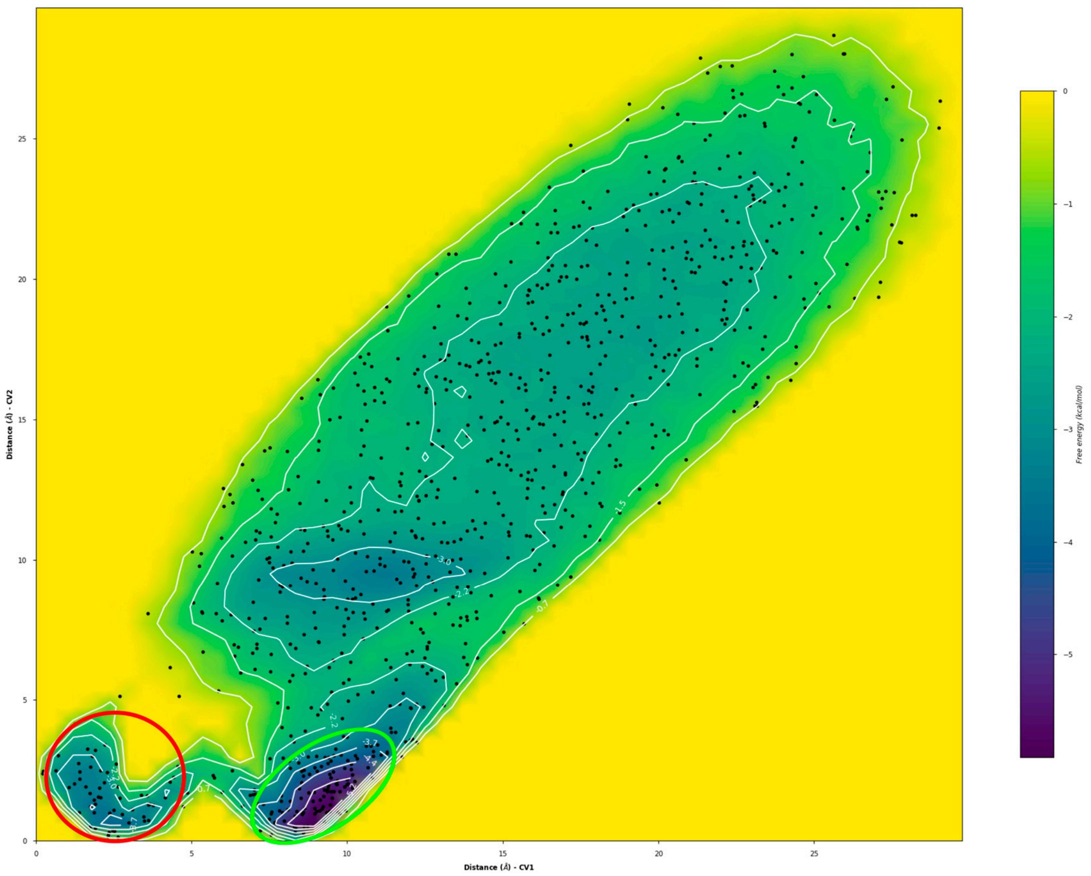

Figure S6. Free Energy Surface from the 240 ns-long metadynamics simulation. Conformation I area is highlighted in green, whilst conformation II area is highlighted in red. Black dots indicate the structures sampled during the simulation.

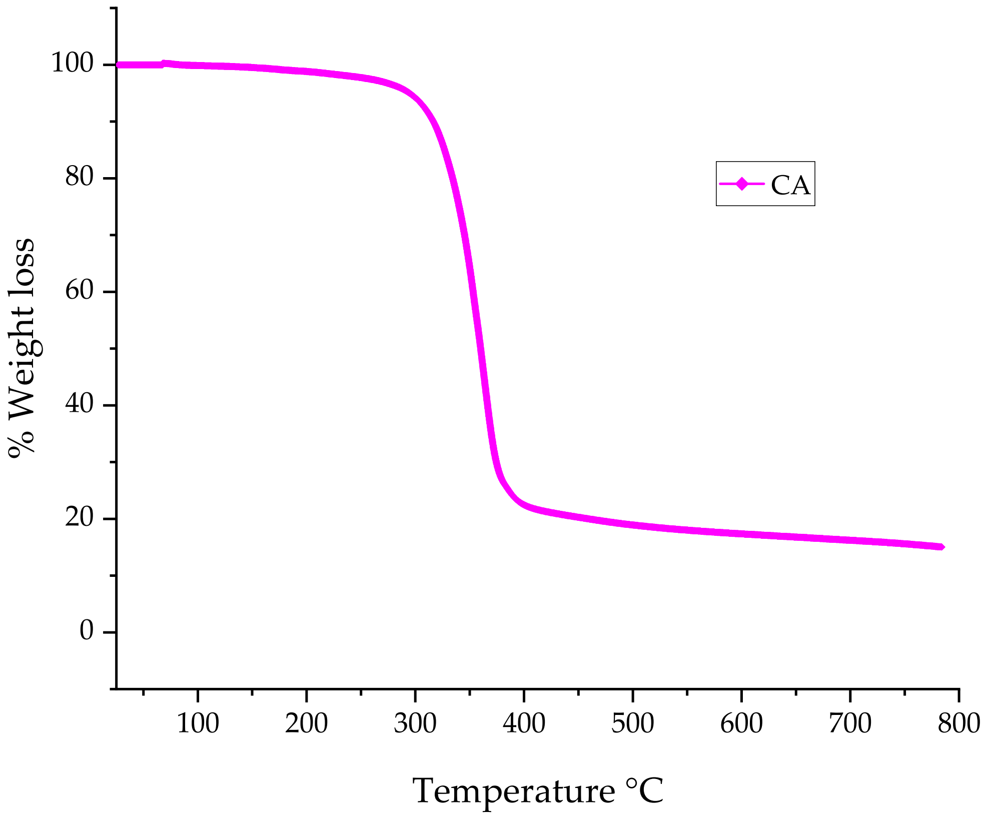

Figure S7. TGA thermograms of CA/RAMEB inclusion complex at 50°C for two months.
